# Differential CARM1 Isoform Expression in Subcellular Compartments and among Malignant and Benign Breast Tumors

**DOI:** 10.1371/journal.pone.0128143

**Published:** 2015-06-01

**Authors:** David Shlensky, Jennifer A. Mirrielees, Zibo Zhao, Lu Wang, Aparna Mahajan, Menggang Yu, Nathan M. Sherer, Lee G. Wilke, Wei Xu

**Affiliations:** 1 Department of Surgery, University of Wisconsin School of Medicine and Public Health, Madison, Wisconsin, United States of America; 2 Department of Oncology, University of Wisconsin School of Medicine and Public Health, Madison, Wisconsin, United States of America; 3 Department of Pathology and Laboratory Medicine, University of Wisconsin School of Medicine and Public Health, Madison, Wisconsin, United States of America; 4 Department of Biostatistics and Medical Informatics, University of Wisconsin School of Medicine and Public Health, Madison, Wisconsin, United States of America; Florida International University, UNITED STATES

## Abstract

**Purpose:**

Coactivator-associated arginine methyltransferase 1 (CARM1) is a coactivator for ERα and cancer-relevant transcription factors, and can methylate diverse cellular targets including histones. CARM1 is expressed in one of two alternative splice isoforms, full-length CARM1 (CARM1FL) and truncated CARM1 (CARM1ΔE15). CARM1FL and CARM1ΔE15 function differently in transcriptional regulation, protein methylation, and mediation of pre-mRNA splicing in cellular models.

**Methods:**

To investigate the functional roles and the prognosis potential of CARM1 alternative spliced isoforms in breast cancer, we used recently developed antibodies to detect differential CARM1 isoform expression in subcellular compartments and among malignant and benign breast tumors.

**Results:**

Immunofluorescence in MDA-MB-231 and BG-1 cell lines demonstrated that CARM1ΔE15 is the dominant isoform expressed in the cytoplasm, and CARM1FL is more nuclear localized. CARM1ΔE15 was found to be more sensitive to Hsp90 inhibition than CARM1FL, indicating that the truncated isoform may be the oncogenic form. Clinical cancer samples did not have significantly higher expression of CARM1FL or CARM1ΔE15 than benign breast samples at the level of mRNA or histology. Furthermore neither CARM1FL nor CARM1ΔE15 expression correlated with breast cancer molecular subtypes, tumor size, or lymph node involvement.

**Conclusions:**

The analysis presented here lends new insights into the possible oncogenic role of CARM1ΔE15. This study also demonstrates no obvious association of CARM1 isoform expression and clinical correlates in breast cancer. Recent studies, however, have shown that CARM1 expression correlates with poor prognosis, indicating a need for further studies of both CARM1 isoforms in a large cohort of breast cancer specimens.

## Introduction

Breast cancer is a heterogeneous disease and is commonly subcategorized based on the expression of intrinsic genomic markers. The most frequently reported markers are the hormone (estrogen and progesterone) receptors [1] as well as the human epidermal growth factor 2 (HER2/neu) [2]. Recently, additional genomic markers have been incorporated into multi-gene platforms such as Oncotype DX, MammaPrint, and Prosigna for prediction of recurrence risk and selection of adjuvant therapies [3]. Increasing interest in personalized cancer care [4] driven by genomic profiling highlights the value of investigating novel biomarkers for the characterization and treatment of breast cancer.

Coactivator-associated arginine methyltransferase 1 (CARM1), a type I protein arginine (R) methyltransferase (PRMT), is one such putative target. CARM1 was originally identified as a coactivator for steroid hormone receptors, including the estrogen receptor (ER), and was later shown to transactivate other cancer-relevant transcription factors including NF-κB, p53, and β-catenin via methyltransferase-dependent and-independent pathways [5]. CARM1 has been shown to methylate histone H3 as well as non-histone proteins including the SWI/SNF core subunit BAF155 [6], CBP/p300 [7], RNA binding proteins, splicing factors [8], and poly-A binding protein-1 [9]. CARM1 knock-out mice die perinatally [10], indicating broad physiological functions in proliferation, differentiation, and development for this coactivator.

CARM1 is overexpressed in a variety of cancer types [11–13], has been identified as an oncogenic client protein of Hsp90 in K562 leukemia cells [14] and regulates tumor metastasis by methylation of BAF155 in MDA-MB-231 breast cancer cells [6]. However, the function of CARM1 in oncogenesis and cancer progression remains unknown, and conflicting evidence supports two opposing roles for CARM1 in proliferation [15–17] and differentiation [11, 18].

The key to reconciling contradictory observations of CARM1 function to date may lie in the expression of distinct alternatively-spliced CARM1 isoforms. Full-length CARM1 (CARM1FL) bears 16 exons, including an automethylation site at exon 15, which is absent in the alternatively spliced product CARM1ΔE15. We have reported that CARM1ΔE15 displays abrogated activation of ERα mediated transcriptional activity and methylates different sets of substrates from those by the full-length CARM1 isoform [19]. Furthermore, CARM1ΔE15 is the predominant isoform in most tissues, while CARM1FL is the major isoform expressed in the luminal compartment of the normal mouse mammary glands [20]. No study to date has directly addressed the functional difference of the two CARM1 isoforms or the significance of differential expression of these isoforms between mammary compartments in human tissues.

It is known that ER expression is more frequently associated with histologically better-differentiated [21], lower grade [22], and less aggressive breast cancers and more favorable disease-free survival [23, 24]. Recent studies suggest that CARM1 expression also correlates with specific sub-cellular compartments that vary by molecular subtype [20] and with clinical outcomes in breast cancer patients. CARM1 expression is associated with poor prognostic factors such as young age of onset, high tumor grade, high proliferation, and increased P-cadherin expression [25]. Given the roles of CARM1 splice isoforms in proliferation and differentiation in breast cancer cells as well as its clinical correlates, CARM1 may be a potential prognostic biomarker. In this study, we found that the two isoforms elicited different sensitivity to Hsp90 inhibitor 17-AAG. The higher sensitivity of CARM1ΔE15 to 17-AAG relative to CARM1FL implicates that this dominant isoform of CARM1 is oncogenic. Consistent with this finding, knockout of CARM1ΔE15 or overexpression of CARM1FL both resulted in growth inhibition, further supporting their opposing roles in regulating cell growth. We used recently developed antibodies to detect differential CARM1 isoform expression in subcellular compartments and among malignant and benign breast tumors.

## Materials and Methods

### Chemicals, Cells and Tissue Culture

17-AAG (Sigma, Cat #A8476) was purchased from Sigma. Human cell lines MDA-MB-231 and BG-1 were purchased from the American Type Culture Collection (ATCC) and used within 3 months. Both cell lines were maintained in Dulbecco’s modified Eagle’s Medium (Life Technologies, Grand Island, NY, USA) with 10% fetal bovine serum (Life Technologies) and were incubated in a humidified atmosphere with 5% CO_2_ at 37°C. CARM1^KO^ MDA-MB-231 cells were generated as previously described [6].

### Human Cancer Tissue Specimens

Twelve flash-frozen human breast cancer tumor samples (6 ER^+^PR^+^HER2^-^; 6 ER^-^PR^-^HER2^-^) and three benign breast tumors (fibroadenoma) were obtained, anonymized and de-identified, from the University of Wisconsin Carbone Comprehensive Cancer Center Translational BioCore BioBank, each of which had been collected at surgical resection. Specimens were processed and flash-frozen within one hour of removal, and kept frozen at -80°C. Acquisition and analysis of samples was conducted according to a protocol certified by the University of Wisconsin Health Sciences Institutional Review Board (IRB 2013–0777).

### Detection of CARM1 Isoform Levels by qRT-PCR

Samples of tumor tissue were homogenized in Trizol (Life Technologies) and RNA was extracted using RNeasy kits (Qiagen) using the automated QiaCube device (courtesy of Dr. Bradfield, McArdle Laboratories, University of Wisconsin). RNA was quantified by nanodrop and 1 μg was added to Superscript II (Invitrogen) for reverse transcription with random primers. qRT-PCR was performed with SYBR Green MasterMix (Invitrogen). Three μL of 1:7 diluted cDNA was added to each 15 μL reaction, and three replicates were performed per specimen. C_t_ values were standardized to the housekeeping gene S18. The primer sequences used were: for human CARM1ΔE15, forward primer: 5’-CAAGGCAGGGGACACG-3’, reverse: 5’-TGGCTGTTGACTGCATAGTG-3’; for human CARM1FL, forward primer: 5’-ATGAGCACGGGGATTGTCCAA-3’, reverse: 5’-TGGCTGTTGACTGCATAGTG-3’. For human 18S rRNA, forward primer 5’ CAGCCACCCGAGATTGAGCA-3’, reverse: 5’-TAGTAGCGACGGGCGGTGTG-3’. The experimenter (DS) was blind to all specimen and patient characteristics (benign v. tumor; molecular subtype; clinical data) during the experiment.

### Immunolabeling of CARM1 in Human Cancer Cell Lines MDA-MB-231 and BG-1

The optimized antibody E15, which only recognizes CARM1FL, and E16, which recognizes both isoforms, were generated and specificity was verified using blocking peptides as previously described [19]. Immunofluorescence: baseline levels of autofluorescence from the secondary antibody were determined by IF using secondary antibody only. Cells were fixed in 4% paraformaldehyde in PBS then blocked in 3% BSA in PBST for twenty minutes, followed by a one hour incubation in primary antibody E15 or E16 (1:300) diluted in 3% BSA, or 3% BSA alone (control). After washing with PBS + 0.1% Triton (PBST), cells were incubated for one hour in secondary antibody goat-anti-rabbit conjugated to FITC (1:1000) diluted in PBST. 50 μL of phalloidin-Alexa 555 were added to each coverslip for fifteen minutes, and then slides were washed with PBST. Fifteen μL of DAPI with Prolong Gold were added to each slide and slides were visualized by fluorescence using a wide-field microscope (LEICA DM500B) with immersion oil on a 100x/1.30 numeric aperture lens. Exposure time, saturation, gamma, gain, and other camera settings were kept constant for images of each fluorophore for each cell line. Fluorescence intensity was measured using Nikon AR Elements software Western blotting of CARM1 was performed as described previously [6].

### Hsp90 Inhibition and Immunoprecipitation

HEK293T cells were transfected with plasmids expressing FLAG-CARM1FL or FLAG-CARM1ΔE15 for 24 hours and then treated with Hsp90 inhibitor, 17-AAG, for 12 hours. CARM1 protein was detected by anti-FLAG antibody in western blot. β-Actin was used as loading control. The protein levels of CARM1 isoforms were quantified using the Odyssey Imaging System. For the immunoprecipitation experiment, HEK293T cells were transfected with plasmids expressing FLAG-CARM1FL or FLAG- CARM1ΔE15 for 24 hours, respectively. The endogenous Hsp90 was immunoprecipitated using α-Hsp90 antibody as described previously [6], and the interacted CARM1 protein was detected by FLAG antibody. Normal rabbit IgG was used as control.

### Immunohistochemistry of CARM1 in Human Breast Samples

Immunohistochemistry staining for tumor tissues was performed on paraffin-embedded sections as previously described [26]. All reagents for IHC were purchased from Biocare Medical (Concord, CA). Briefly, following de-paraffinization and antigen retrieval, endogenous peroxidase was blocked with Peroxidazed I, then Background Punisher was added. After Avidin-Biotin blocking for 15 minutes each, tissue sections were incubated with rabbit anti-CARM1 E15 (1:1000) and E16 (1:2000) primary antibodies (GeneMed) for 2 hours at room temperature. Following this, goat anti-rabbit biotinylated secondary IgG antibodies were applied for 15 minutes at room temperature. Streptavidin-HRP was applied for another 15 minutes before staining with Betazoid DAB and then tissue sections were counterstained with CAT hematoxylin.

### Statistical Analyses

All data were analyzed in SPSS (IBM, Inc.) by ANOVA. Significant results from ANOVA were further analyzed post-hoc by Tukey’s. Relationships between continuous variables were determined using bivariate correlations with Pearson’s R. Analyses were considered significant if p ≤ 0.05.

## Results

### Cytoplasmic localization of CARM1ΔE15 and CARM1FL

**Fig 1:**
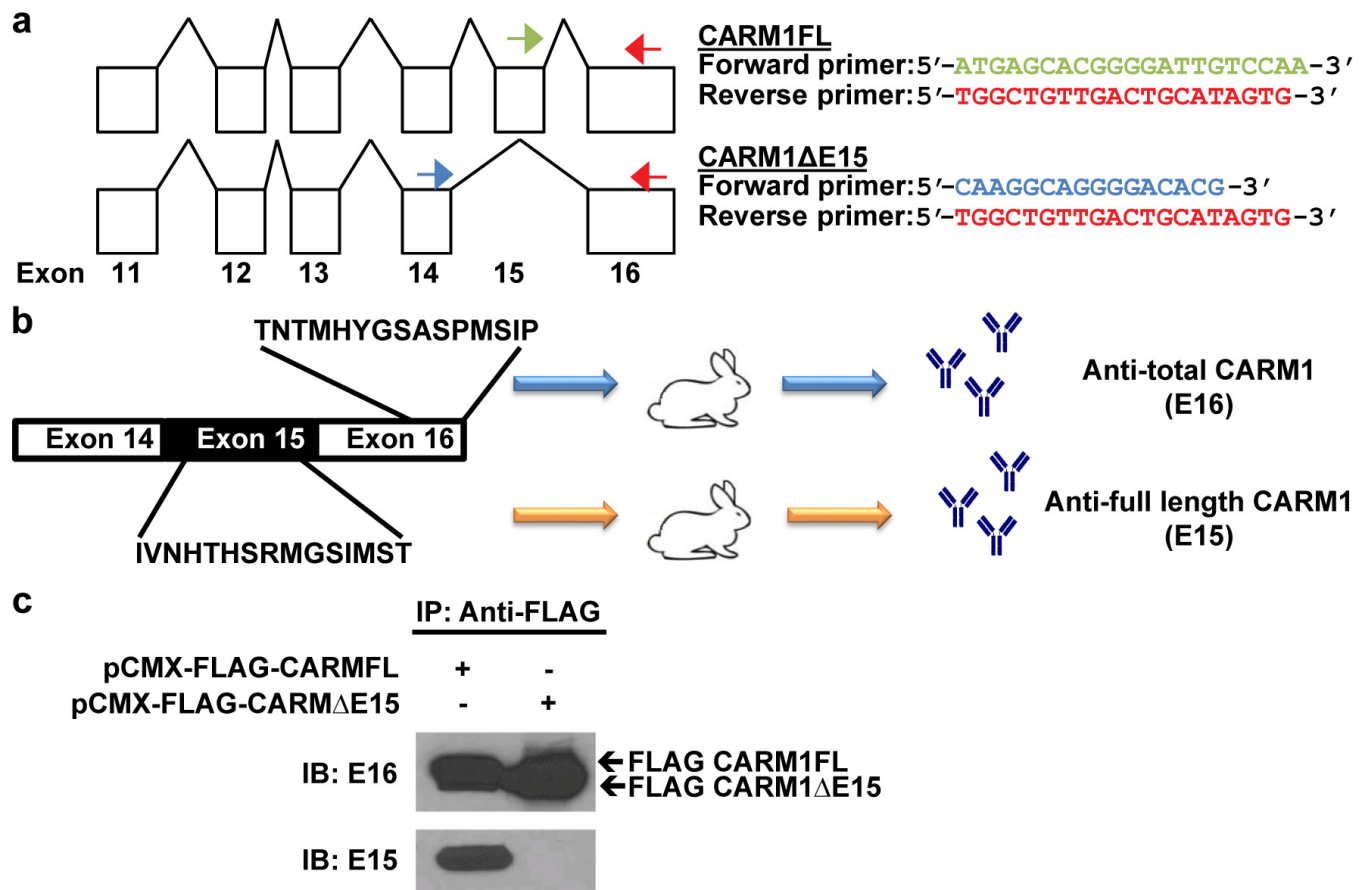
Detection of CARM1 isoforms. (a) Sequences of primers designed against CARM1FL and CARM1ΔE15. (b) Two CARM1 rabbit polyclonal antibodies were generated using indicated peptide sequence as antigens. (c) Western blotting result shows that E16 detects total CARM1 and E15 detects only CARM1FL. IB: immunoblot. IP: immunoprecipitation.

We have previously shown that CARM1 exists in two isoforms, which can be detected at the level of mRNA via PCR (Fig 1A) or by antibodies specifically developed for CARM1FL (E15) or both isoforms (E16) against the indicated targeted sequences (Fig 1B). In order to study the subcellular localization of CARM1 isoforms using E15 and E16 antibodies, we first characterized antibody specificity. FLAG-tagged CARM1FL or CARM1ΔE15 expressing plasmids were transiently transfected into HEK293T cells. CARM1 was precipitated using anti-FLAG antibody and detected using E15 or E16 antibodies. Western blotting showed that as expected, E15 only recognizes CARM1FL and E16 could detect both isoform proteins (Fig 1C).

**Fig 2:**
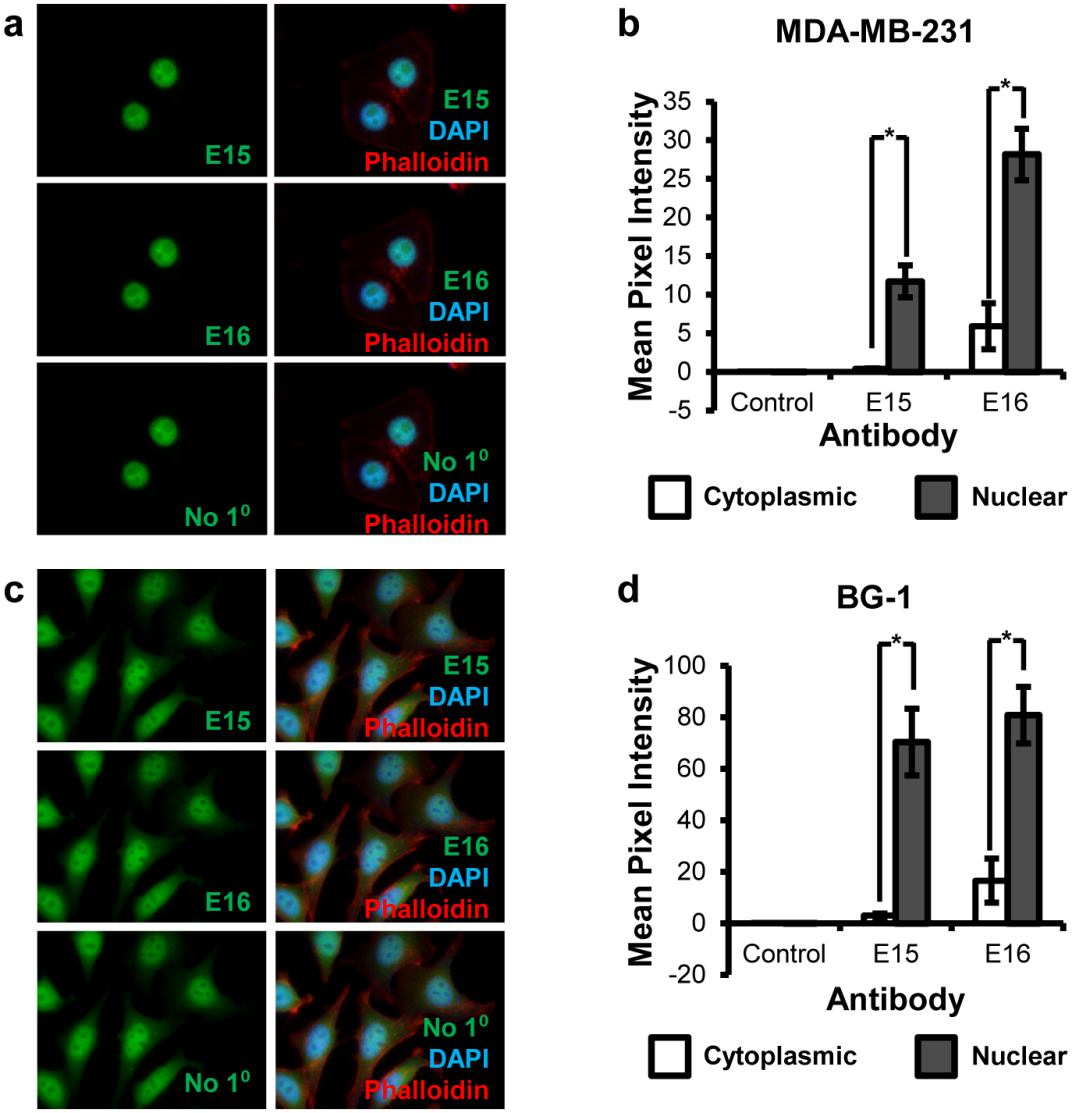
Immunofluorescence analysis of CARM1 localization in human cell lines. (a, b) Localization of CARM1FL (E15) and total CARM1 (E16) in MDA-MB-231 cells. (c, d) Localization of CARM1FL (E15) and total CARM1 (E16) in BG-1 cells. (b) and (d) are quantification of immunofluorescence signals in cytoplasm and nucleus. DAPI: nuclear stain (blue). Phalloidin: cytoskeleton/actin probe (red). Student’s t test was used for statistical analysis. n = 3, *p < 0.05.

We next used these antibodies for immunofluorescence staining to determine subcellular localization of both isoform proteins in two cancer cell lines, MDA-MB-231 and BG-1. Both E15 and E16 antibodies detected CARM1 in both cytoplasm and nucleus (Fig 2A). Quantification of the immunofluorescence intensity in MDA-MB-231 breast cancer cells showed that the ratio of cytoplasmic fluorescence to total fluorescence was higher in E16 stained cells (15.75%) than E15 stained cells (3.39%; p < 0.005). Cytoplasmic E15 intensity was not significantly above background (p = 0.357); however, nuclear E15 (p = 0.002), nuclear E16 (p < 0.005), and cytoplasmic E16 (p < 0.005) staining were all significantly higher than secondary antibody only controls (Fig 2B). These results indicate that CARM1ΔE15 accounts for the majority, if not all of the cytoplasmic staining of CARM1 by E16 antibody.

The immunofluorescence experiment was next performed on BG-1 cells, an ER+ ovarian cancer cell line, to verify CARM1 isoform localization was not cell line or cancer-type specific. Similar to the results from MDA-MB-231 cells, the two antibodies detect CARM1 in both cytoplasm and nucleus (Fig 2C) yet with different ratio. The percentage of cytoplasmic fluorescence to total fluorescence was significantly higher in E16 stained cells than E15 stained cells (mean = 16.73% and 3.80%, respectively; p < 0.005). Similarly, cytoplasmic E15 intensity was not significantly different from secondary antibody control cytoplasmic intensity (p = 0.093), indicating a low level of CARM1FL in cytoplasm, whereas nuclear E15 (p < 0.005), nuclear E16 (p < 0.005), and cytoplasmic E16 (p < 0.005) staining were all significantly higher than their respective controls (Fig 2D). Cells were not significantly different in size between each antibody group in either MDA-MB-231 or BG-1 cells (p = 0.42 and p = 0.78, respectively). In both cell lines, CARM1ΔE15 was consistently identified as the major cytoplasmic form, whereas CARM1FL is more nuclear localized.

### Characterization of CARM1 Isoform Functional Differences by Hsp90 Sensitivity and Growth Modulation Effects

**Fig 3:**
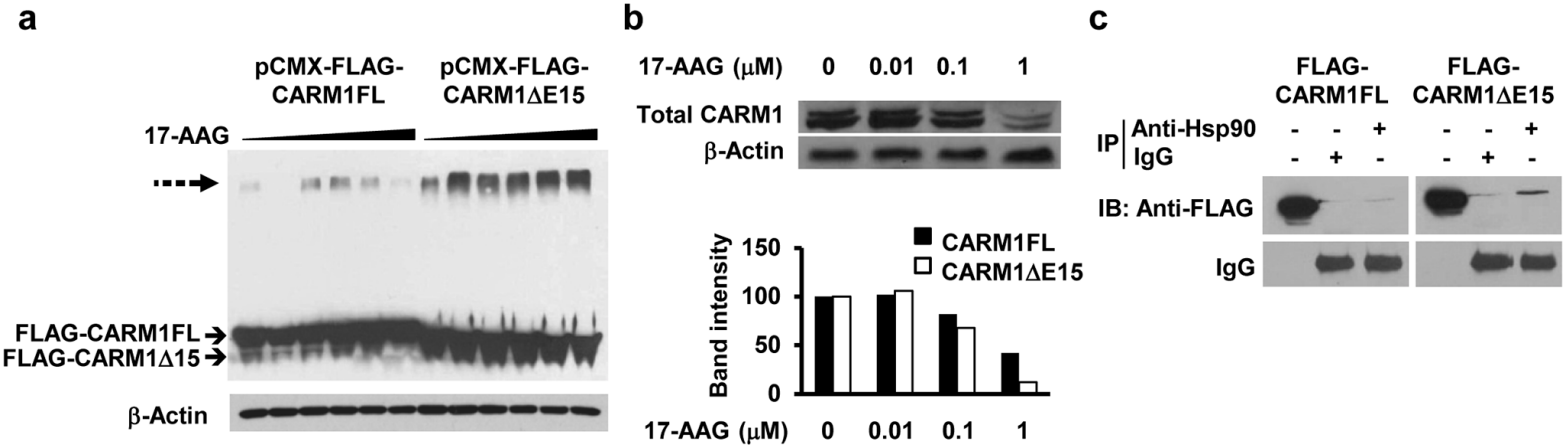
Characterization of CARM1 isoform sensitivity to Hsp90. (a) Hsp90 inhibition of HEK293T cells transfected with FLAG-CARM1FL or FLAG-CARM1ΔE15. CARM1 protein was detected by anti-FLAG antibody. Dotted arrow indicates polyubiquitinated CARM1. (b) Effect of Hsp90 inhibition on endogenous CARM1FL and CARM1ΔE15 in HEK293T cells detected by western blot (upper panel) and quantified (lower panel). (c) FLAG-CARM1FL and FLAG-CARM1ΔE15 both co-immunoprecipitate with Hsp90. IB: immunoblot. IP: immunoprecipitation. β-Actin: loading control.

**Fig 4:**
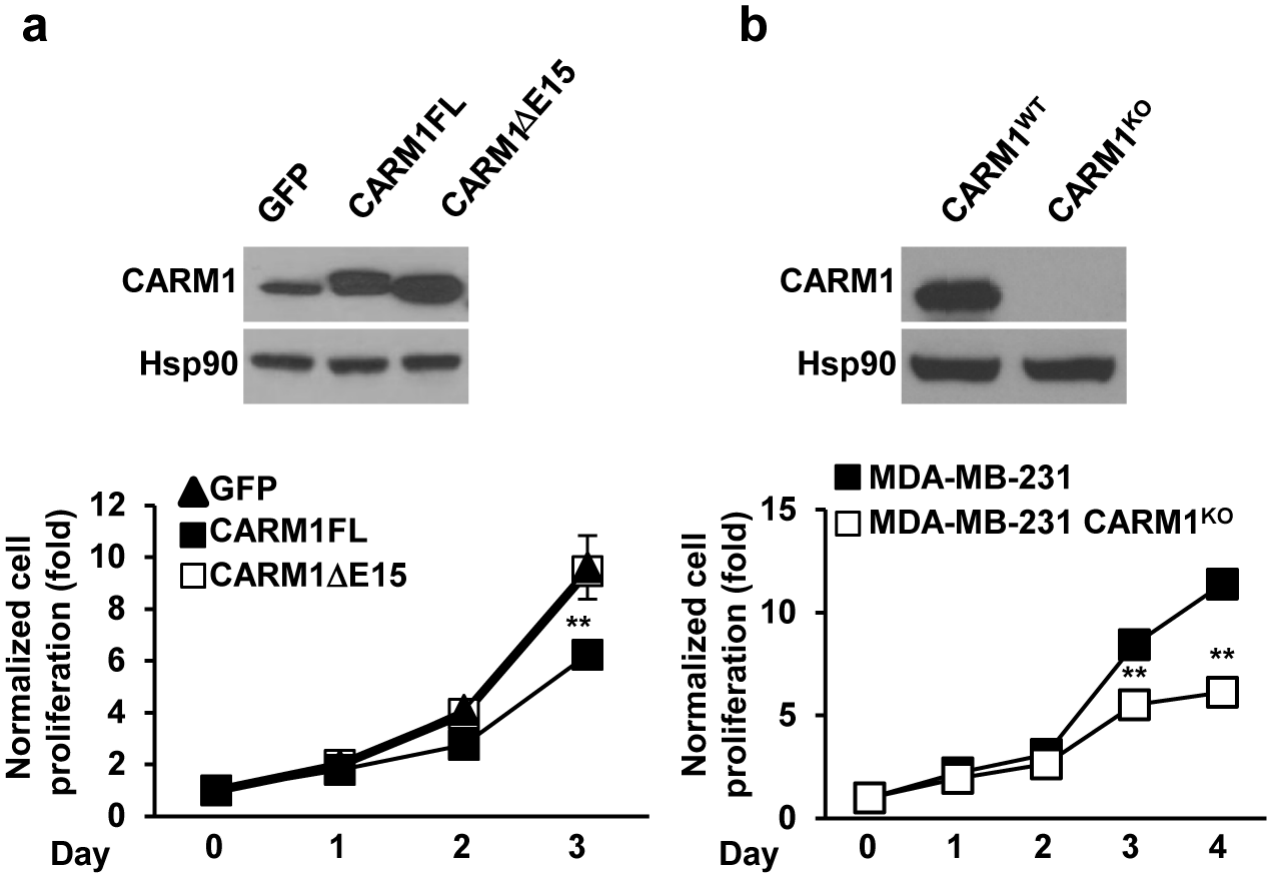
Differential effects of CARM1FL and CARM1ΔE15 expression on cell growth in vitro. (a) MDA-MB-231 cells were infected with retrovirus expressing GFP, CARM1FL and CARM1ΔE15, respectively. The protein level of CARM1 was detected by western blot (upper panel). The cell proliferation rate was determined by MTT assay (lower panel) [6]. (b) The protein level of CARM1 was detected by western blot (upper panel). The cell proliferation rate was determined by MTT assay (lower panel). ** p < 0.01.

It has previously been shown that oncogenic clients of Hsp90 proteins are more sensitive to Hsp90 inhibitors, resulting in polyubiquitinylation and degradation. CARM1, in turn, is a reported oncogenic client of Hsp90 [14, 27]. To experimentally determine if the two CARM1 isoform proteins exhibit different sensitivity to Hsp90 inhibitors, plasmids expressing FLAG-tagged CARM1FL or CARM1ΔE15 were transiently transfected to HEK293T cells and the cells were treated with increasing doses of an Hsp90 inhibitor, 17-AAG (Fig 3A and 3B). While both CARM1 isoforms were polyubiquitinated proportionally to the amount of inhibitor, stronger staining of polyubiquitinated CARM1 was detected in CARM1ΔE15 expressing cell lysates as compared with CARM1FL expressing cell lysates, indicating that CARM1ΔE15 is more prone to degradation upon treatment with 17-AAG. At 1 μM of 17-AAG, CARM1ΔE15 protein levels were significantly more inhibited than CARM1FL levels (Fig 3B). Co-immunoprecipitation data demonstrates that the interdependence of CARM1 and Hsp90 may be mediated via direct protein-protein interaction (Fig 3C). In a separate experiment in MDA-MB-231 cell lines, two-fold overexpression of CARM1FL resulted in diminished cell counts in populations transfected with vectors for the expression of CARM1FL (p < 0.01; Fig 4A), in contrast, overexpression of CARM1ΔE15 or a GFP control did not lead to a significantly different rate in cell growth. Conversely, as we previously reported [6], knock-out of CARM1 by zinc finger in MDA-MB-231 which expresses predominately CARM1ΔE15 decreased cell numbers and inhibited growth as compared with the parental cells. These changes were significant after day 3 (p < 0.01; Fig 4B).

### CARM1 Isoform Expression and Patient Clinical Characteristics

**Fig 5:**
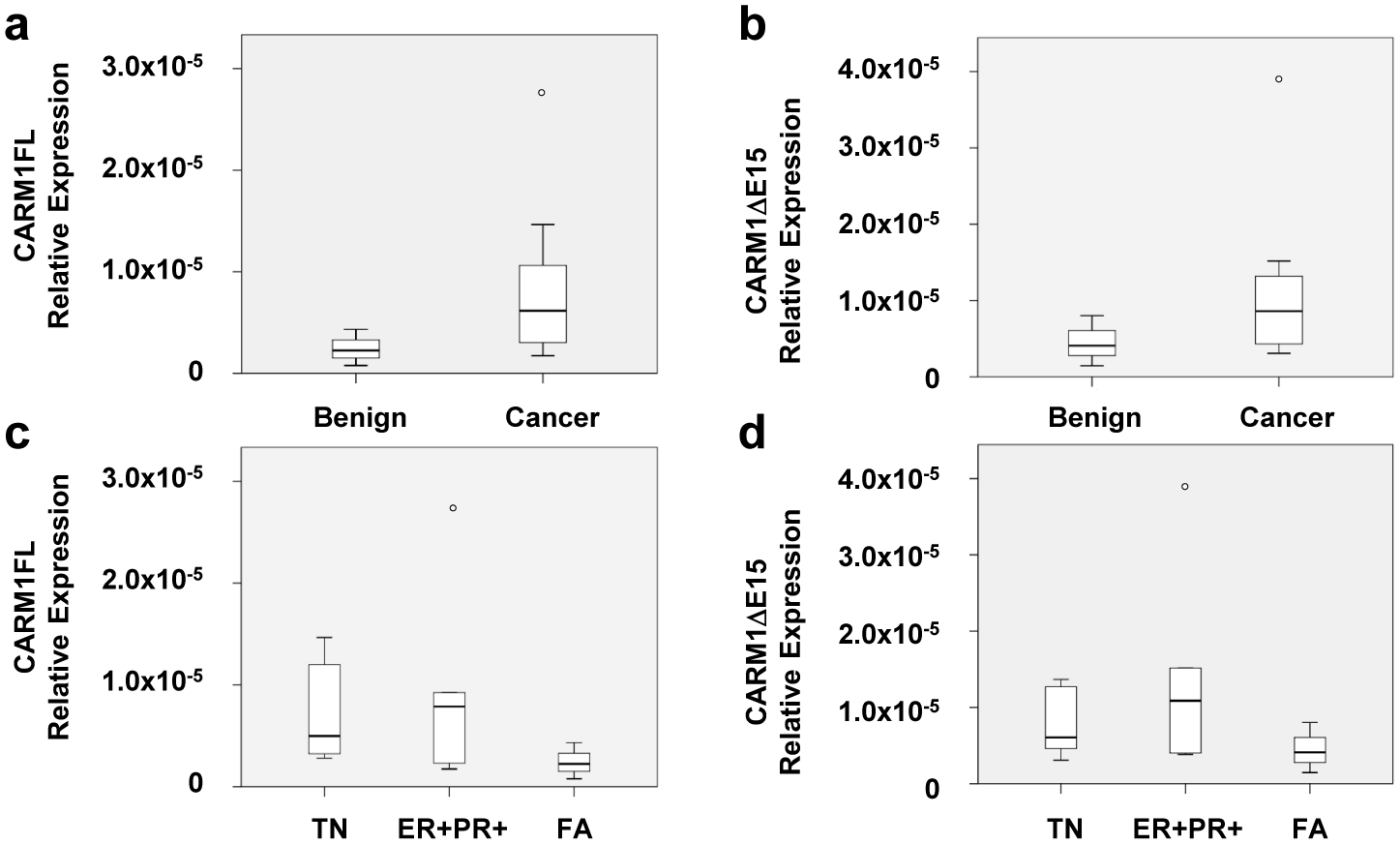
qPCR analysis of CARM1FL and CARM1ΔE15 isoforms in primary breast samples: tumor type and receptor status. Tumor type and receptor status are not correlated with CARM1FL (a, c) or CARM1ΔE15 (b, d) mRNA levels. Tumor types: triple negative (TN), estrogen receptor positive and progesterone receptor position (ER+PR+), benign fibroadenoma (FA).

**Fig 6:**
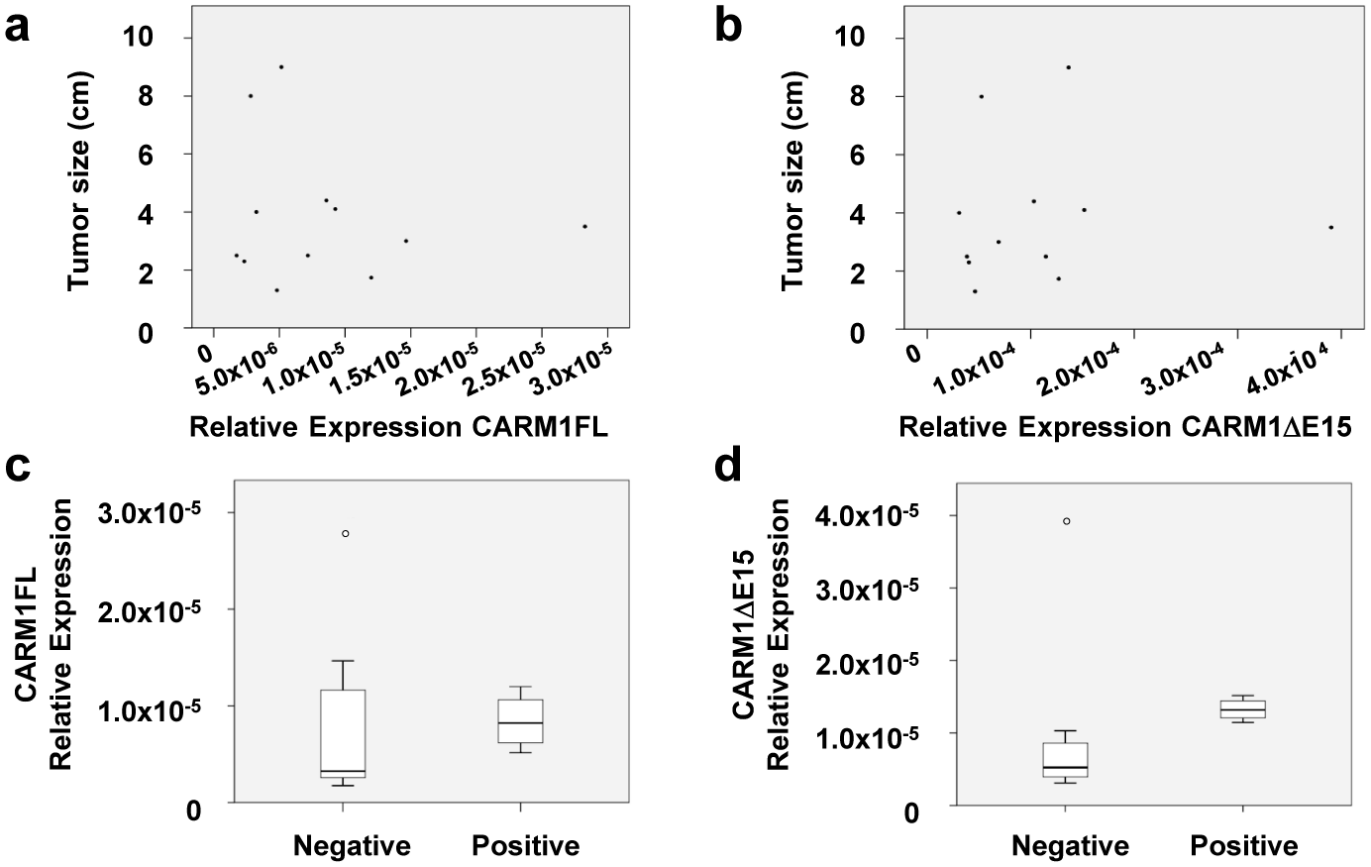
qPCR analysis of CARM1FL and CARM1ΔE15 isoforms in primary breast samples: tumor size and lymph node status. (a, b) Tumor size and (c, d) lymph node status are not correlated with CARM1FL or CARM1ΔE15 mRNA levels.

To determine the association of CARM1 isoform levels with patient clinical characteristics, we processed twelve breast cancer tumors and three benign fibroadenoma tumors for qRT-PCR using validated primers (Table 1). Cancer samples did not have significantly higher expression of CARM1FL or CARM1ΔE15 mRNA compared to benign fibroadenomas (p = 0.21 and p = 0.31, respectively; Fig 5A and 5B), even when stratified by hormone receptor status (p = 0.39, p = 0.30, p = 0.308, for CARM1FL, CARM1ΔE15, and total CARM1, respectively; Fig 5C and 5D). CARM1FL levels did not correlate with tumor size (R = -0.159; p = 0.621; Fig 6A and 6B). Tumors from patients with lymph node involvement did not have significantly higher expression of CARM1FL (p = 0.94) nor CARM1ΔE15 (p = 0.67) (Fig 6C and 6D). Finally, no correlation was found between CARM1ΔE15 expression levels and tumor size (R = 0.06; p = 0.85) or the number of positive lymph nodes (p = 0.64) in cancerous samples.

**Table 1:** Patient clinical characteristics.

Histologic diagnosis*	
Invasive ductal carcinoma	7/15 (46.7%)
Invasive lobular carcinoma	3/15 (20.0%)
Adenosquamous carcinoma	1/15 (6.7%)
Infiltrating micropapillary carcinoma	1/15 (6.7%)
Benign	3/15 (20.0%)
Receptor status**	
ER+PR+HER2-	6/12 (50.0%)
ER-PR-HER2-	6/12 (50.0%)
Histological grade**	
0	1/12 (8.3%)
1	1/12 (8.3%)
2	3/12 (21.4%)
3	7/12 (42.8%)
Stage**	
T1	1/12 (8.3%)
T2	9/12 (75.0%)
T3	2/12 (16.7%)
Lymph node status**	
Negative	7/12 (58.3%)
Positive	4/12 (33.3%)
N/A (no LN surgery)	1/12 (8.3%)
Neoadjuvant chemotherapy**	
No	11/12 (91.7%)
Yes	1/12 (8.3%)

*All samples.

**Cancer samples only. ER: estrogen receptor.

PR: progesterone receptor. HER2: human epidermal growth factor receptor 2. LN: lymph node. N/A: not applicable.

**Fig 7:**
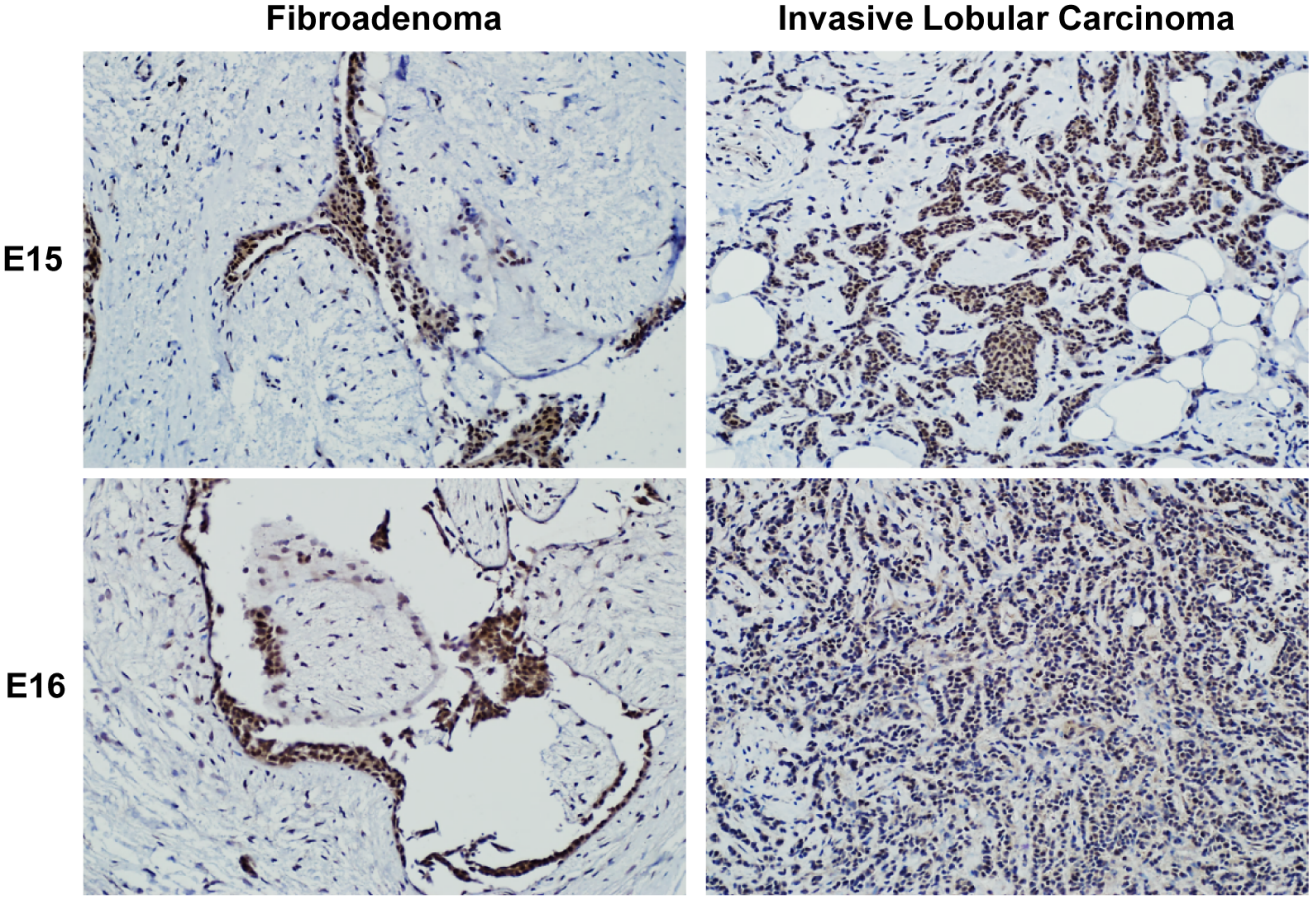
A representative immunohistochemistry staining image of CARM1 isoforms in primary breast sample. Benign (fibroadenoma) and cancer (invasive lobular carcinoma) clinical samples were stained with E15 and E16 antibodies to assess CARM1 subcellular localization. Images captured at 20X magnification.

We additionally examined CARM1 protein expression in fibroadenoma and invasive lobular carcinoma breast tissue samples representing benign and cancer tissues respectively using immunohistochemistry (Fig 7). Both CARM1FL (E15 antibody) and total CARM1 (E16 antibody) are preferentially expressed in the epithelial cells, with little stromal expression of either isoform. Strong nuclear E15 positivity was observed in both the fibroadenoma and the invasive lobular carcinoma, with weak cytoplasmic E15 staining. E16 signal was observed in the nuclear and cytoplasmic compartments of both samples, with stronger signal in the nucleus than in the cytoplasm.

## Discussion

CARM1 plays opposing roles in proliferation and differentiation in breast cancer cells through the expression of two splice isoforms, CARM1FL and CARM1ΔE15. These isoforms feature different activities in ERα co-activation, substrate methylation, and distribution in mammary epithelial and stromal cells [19]. Previous research appeared to support the argument that CARM1 is oncogenic in that the reduction of CARM1 expression decreased E2F1 levels and cell cycle progression in MCF7 breast cancer cells [17]. Because MCF7 is a cell line in which CARM1ΔE15 accounts for greater than 85% of the expressed CARM1 [19], this conclusion does not represent the whole story. Our aforementioned experiments replicate the results in Frietze et al. [17] by showing that CARM1 knock out in MDA-MB-231 cells, another line in which greater than 85% is CARM1ΔE15 [19], decreases cell division. Furthermore, we expand on these findings by showing that overexpression of CARM1FL in the same cell line acts in an opposite manner and decreases cell division. This finding is in conformity with a previous report of cell growth inhibition when full-length CARM1 is inducibly or stably expressed in MCF7 cells [18]. These seemingly contradictory results between studies [17, 18] merely support a new paradigm in the analysis of CARM1 that emphasizes isoform specific differences. In particular, CARM1 is likely to play either an oncogenic or inhibitory role depending on which isoform is dominant in the cell population.

These findings are in agreement with the observation that CARM1ΔE15 is more sensitive to Hsp90 inhibition than CARM1FL, indicating that the truncated isoform may be the oncogenic form [14]. It should be noted that in multiple breast cancer cell lines regardless of molecular subtype, CARM1ΔE15 is the dominant endogenous isoform [19]. Future studies should thus utilize targeted expression of each isoform in CARM1KO cells to allow for the improved delineation of their respective functions.

In addition to *in vitro* data that supports that CARM1 isoforms are important cancer related proteins, several previous studies have suggested that overall CARM1 expression is related to oncogenesis and poor outcomes in human breast cancer tissues. A positive correlation has been observed between CARM1 and PELP1, another transcriptional coregulator of nuclear receptors, in non-Luminal A tumors, and high expression of both strongly correlates with proliferative marker expression [28]. Habashy et al [25] reported that CARM1 expression is correlated with poor prognostic factors such as young age of onset, high tumor grade, high proliferation, increased basal cytokeratins and P-cadherin expression, and p53 mutations. Moreover, a positive association was found between CARM1 protein expression and EGFR family members. The patients whose tumors are in CARM1^high^, HER2^+^ category displayed a worse survival than those in CARM1^low^, HER2^+^ category, indicating a possible crosstalk between EGFR family members and CARM1. Consistent with this report, Cheng et al also observed strong correlation of expression between CARM1 and HER2 in 247 tumor specimens derived from the Chinese women [29]. In addition, increased CARM1 expression was observed in both nucleus and cytoplasm in breast invasive carcinoma as compared with the matched benign tissues adjacent to the tumors [29]. The cytoplasmic and nuclear staining of CARM1 was also observed in a large tissue microarray study using over 800 histological samples derived from 549 US and African patients [20]. Interestingly, this study reported that higher nuclear CARM1 levels are associated with HER2 status, whereas higher cytoplasmic CARM1 are associated with basal-like triple negative subtype, which typically are associated with the worst outcome [20]. Interestingly, increased cytoplasmic versus nuclear CARM1 levels was found in African women relative to women with African American and Caucasian ethnicity [20]. It is worthy to note that all studies referenced here used different CARM1 antibodies for immunohistochemistry and different patient ethnicity pools. Nonetheless, overexpression of CARM1 was consistently found associated with poor prognosis and CARM1 could be in both cytoplasm and nucleus. In line with these findings, by employing two antibodies recognizing total and full-length CARM1 proteins, our data indicate that *in vitro* CARM1ΔE15 is likely a larger contributor to cytoplasmic CARM1. Despite these localization differences, we failed to find an association between the expression of either CARM1 isoform with malignancy, molecular subtype, tumor size, or lymph node status. The discrepancy may be attributed to the fact that the previous studies did not take into account of the differential isoform expression and the CARM1 antibodies used in this study are different from all other published studies. One limitation of this study, however, is the sample size. The analysis presented in this study included only 15 tumor specimens. More solid understanding of the relationship between cytoplasmic or nuclear CARM1 with clinicopathologic parameters warrants the use of CARM1 immunostaining using a larger tissue microarray.

Another factor that remains to be further examined is the distinction between epithelial CARM1 and stromal CARM1. Normal mouse mammary stroma is enriched in CARM1ΔE15 [19]. It has been reported previously that CARM1 is enriched in HER2^+^ breast cancer [20]; however, the localization of CARM1 expression in the epithelial and stromal compartments was not described in that study. In the absence of microdissection to isolate epithelia and stroma, background expression of either isoform in the stroma could serve as a significant confounding factor for the interpretation of CARM1 expression in cancerous epithelia. The qPCR experiments reported here are subject to this caveat, and it must be noted that our experimental methodology did not allow for differentiation of stromal and epithelial CARM1 expression. Our immunohistochemical analysis of one benign and one malignant breast sample, however, did not indicate significant stromal expression of either isoform. Furthermore, pathological examination of one of our reported malignant samples revealed only adjacent normal background breast tissue, indicating a need for improved methodology in the preparation of clinical samples linked to correct pathological descriptors in order to answer questions about the independent role of each CARM1 isoform in cancer progression. Future studies employing parallel qPCR and immunohistochemistry on a larger set of samples will be necessary to more accurately assess whether CARM1 isoform levels are indeed related to patient clinical characteristics.

In this study, we did not find a correlation between the expression of either CARM1 isoform with molecular subtype or clinical disease characteristics among our cohort of breast cancer patients and women with benign breast lesions. However, our analysis of CARM1FL and CARM1ΔE15 in MDA-MB-231 cells revealed key functional and stability differences indicating distinct roles for each isoform in breast cancer cell proliferation. The sensitivity of CARM1ΔE15 to the Hsp90 inhibitor 17-AAG and the growth inhibitory phenotype when CARM1 was knocked out in CARM1ΔE15 expressing cells indicate that this isoform may be the oncogenic form. Localization that favors CARM1ΔE15 dominance in the cytoplasm is in line with expectations provided by the other research [20] and also provides plausibility to a relationship between CARM1ΔE15 with cancer progression *in vivo*. Even though these differences were not elucidated in our tissue based experiments, these findings support a revised paradigm regarding CARM1 in which CARM1ΔE15 supports oncogenesis, whereas CARM1FL might have a protective role. No previous studies have paid attention to these two isoforms as separate contributors. Thus, further elucidation of the role of CARM1 in breast cancer will require experiments that also take into account functional differences between these two related, yet distinct players in human cancers.
