# Correction: Differential CARM1 Isoform Expression in Subcellular Compartments and among Malignant and Benign Breast Tumors

**DOI:** 10.1371/journal.pone.0131955

**Published:** 2015-06-25

**Authors:** David Shlensky, Jennifer A. Mirrielees, Zibo Zhao, Lu Wang, Aparna Mahajan, Menggang Yu, Nathan M. Sherer, Lee G. Wilke, Wei Xu

There are errors in panels A and C of Fig 2. The authors have provided a corrected version of Fig 2 here.

**Fig 2:**
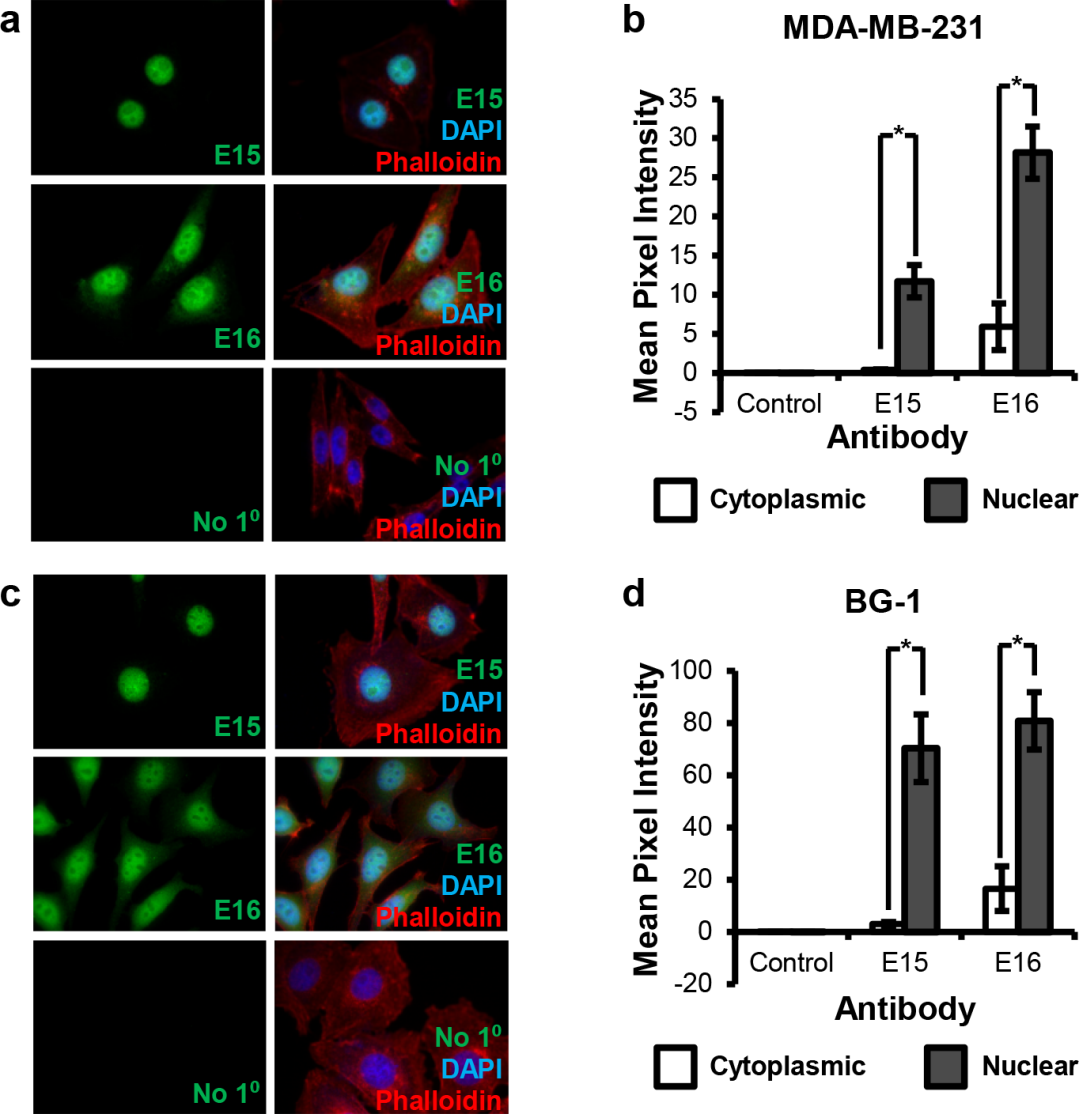
Immunofluorescence analysis of CARM1 localization in human cell lines. (a, b) Localization of CARM1FL (E15) and total CARM1 (E16) in MDA-MB-231 cells. (c, d) Localization of CARM1FL (E15) and total CARM1 (E16) in BG-1 cells. (b) and (d) are quantification of immunofluorescence signals in cytoplasm and nucleus. DAPI: nuclear stain (blue). Phalloidin: cytoskeleton/actin probe (red). Student’s t test was used for statistical analysis. n = 3, *p < 0.05.
